# Placental glucose transporter 1 and 3 gene expression in Monochorionic twin pregnancies with selective fetal growth restriction

**DOI:** 10.1186/s12884-021-03744-2

**Published:** 2021-03-27

**Authors:** Yao-Lung Chang, An-Shine Chao, Shuenn-Dyh Chang, Po-Jen Cheng

**Affiliations:** Department of Obstetrics and Gynecology, Chang Gung Memorial Hospital, Linkou medical center and College of Medicine, Chang Gung University, Taoyuan, Taiwan

**Keywords:** Glucose transporter 1, Glucose transporter 3, Hypoxia-inducible factor-1α, Monochorionic twin, Fetal growth restriction, Placenta

## Abstract

**Background:**

In monochorionic twin (MC) gestations with selective fetal growth restriction (FGR), the discordant fetal growth usually is due to unequal placental sharing. Glucose, which is essential for oxidative metabolism in the growing placenta and fetus, is transferred from maternal blood by facilitated carrier-mediated diffusion via glucose transporters (GLUTs). How the GLUTs expression varies in the two placenta territories manifests discordant perfusion in MC twin pregnancy with selective FGR is unknown. This study evaluates the human placental GLUT1 and GLUT3 gene expression in MC twin gestations with selective FGR.

**Methods:**

MC twin pregnancy with selective FGR was defined as the presence of inter-twin birth weight discordance of > 25% and the smaller twin with a birth weight less than the 10th percentile in third trimester. Fetal umbilical artery Doppler was checked within 1 week before delivery in the two fetuses. An abnormal umbilical artery Doppler was defined as persistently absent or reverse end-diastolic flow (UA-AREDF). GLUT1, GLUT3 and HIF-1α gene expression were assayed in each twin’s placental territories. The inter-twin placental gene expression ratio was calculated as the placenta GLUTs or HIF-1α expression level of the selective FGR twin divided by expression level of the appropriate-for-gestational-age (AGA) cotwin. Higher gene expression ratio means elevated gene expression in the selective FGR twin’s placenta territory compared to AGA twin’s placenta territory.

**Results:**

15 MC twin gestations with selective FGR including nine with normal (group 1) and six with abnormal selective FGR twin UA Doppler (group 2) were included into this study. The GLUT3 and HIF-1α gene expression are significantly elevated in selective FGR twin’s placenta territory in group 2 twin pregnancies (mean gene expression ratio as 2.23 and 1.65, *p* values as 0.015 and 0.045, respectively), but not in in group 1 twin pregnancies.

**Conclusion:**

The upregulation of placental GLUT3 gene expression in selective FGR fetus with abnormal UA Doppler may be due to hypo-perfusion which is mediated by up -regulation of HIF-1α gene expression.

## Background

Fetal growth restriction (FGR) is defined as a rate of fetal growth that is less than normal for the growth potential of a specific infant [1]. FGR fetus may be not only with increasing risk of complications immediately after delivery like perinatal asphyxia, renal insufficiency and pulmonary hemorrhage; but also with long term complications like increased risk of diabetes mellitus and hypertension in adult life [2], which call fetal origin of adult disease. Barker hypothesized the fetal origin of adult disease as that those FGR fetuses would adapts to a poorer intrauterine environment by optimizing the use of a reduced nutrient supply to ensure survival [3]. These changes might aid survival of the fetus, but they are a liability in situations of nutritional abundance, they would have a higher chance to get metabolic disease like type II diabetes mellitus [4]. But the true mechanism of born as small and later more risk of adult disease is still unclear. Glucose is essential for oxidative metabolism in the growing placenta and fetus; it is transferred from maternal blood to placenta and fetus by facilitated carrier-mediated diffusion via glucose transporters (GLUTs). Among the isoforms of the GLUTs: GLUT-1, GLUT-3, GLUT-4, GLUT-8, GLUT-9 and GLUT-12 proteins and the mRNA of GLUT-10 and GLUT-11 have been detected in the human placenta [5], among them, GLUT1, GLUT3 and GLUT 4 have been reported as the primary transport proteins [6, 7]. GLUT4 is an insulin-sensitive isoform, which may mediate insulin-stimulated placental glucose uptake in early pregnancy [8], which may not play as an important role as GLUT1 and GLUT3 at advanced gestational age.

According to Barker theory, the glucose utilization in FGR fetus during intrauterine life is different from appropriate for gestational age fetus [4, 9], so the glucose transferred from maternal circulation to FGR fetus may not proportionally reflect their body weight discrepancy. Study results of placental GLUTs in human pregnancy affected by FGR have been conflicting [10]. Among the FGR studies in singleton pregnancies, there was a lack of agreement on the definitions of FGR [11, 12]. Comparison groups (non FGR) were from the general population with genetic differences; different fetal weight may well be caused by genetic factors. A twin pregnancy with selective FGR is defined as one FGR twin with an appropriate for gestational age (AGA) cotwin, because the AGA twin can achieve the genetically determined birth weight, the selective FGR fetus likely represents true growth restriction. In this model, the AGA twin’s placental area GLUTs expression can be used to compare with the FGR twin’s placenta area GLUTs expression. Thus, a twin pregnancy with selective FGR represents a suitable study object to elucidate the connection between placenta GLUTs expressions and FGR.

Additionally GLUT1 and GLUT3 gene expression were reported as upregulated in primary syncytotrophoblast cells upon exposure to hypoxia [6]. Hypoxic upregulation of glucose transporters in BeWo choriocarcinoma cells is mediated by hypoxia-inducible factor-1α (HIF-1α) [13]. The aim of this study is to investigate the GLUT1, GLUT3 and HIF-1α gene expression in both fetuses’ placental territories in MC twin pregnancy with selective FGR.

## Materials and methods

This study was conducted from September 2019 to September 2020 at Chang Gung Memorial Hospital, Linkou Medical Center, Taiwan. The study protocol was approved by the Institutional Review Board of the Chang Gung Medical Foundation (IRB # 201802017A3). The inclusion criteria required MC twin pregnancies delivered in our hospital, mothers with MC twin pregnancies provided with written informed consents, and placental tissues successfully collected. The exclusion criteria were the presence of twin-to-twin transfusion syndrome (TTTS), monoamniotic twins, twin anemia–polycythemia sequence (TAPS), and congenital, structural or genetic malformations in the fetus. To assure the placentas would be intact, only MC twins gestations delivered through cesarean section were included to ensure an intact placenta.

TTTS was diagnosed according to the Quintero criteria [14] and TAPS was diagnosed as postnatal inter-twin hemoglobin difference of > 8 g/dL [15]. MC twin pregnancies were identified in the first trimester or early second trimester using the following ultrasonographic criteria: (I) the presence of a single placenta, (II) the presence of a thin dividing membrane, and (III) the absence of a twin peak (lambda) sign. Monochorionicity also was confirmed by obstetricians through postpartum examination of the placenta (presence of a single placenta with inter-twin anastomoses).

Selective FGR was defined as the presence of (I) a birth weight discordance of > 25% and (II) an FGR twin with a birth weight less than the 10th percentile [16, 17]. Birth weight discordance was calculated as the difference between the fetal weights of the AGA and FGR twins divided by the fetal weight of the larger twin as follows: [(body weight of the AGA twin − body weight of the selective FGR twin)/(body weight of the AGA twin)] × 100%.

### Inspection and collection of placental tissues

The placentas were cut along the vascular equator. We collected two to three pieces of fresh placental tissue (2 × 2 × 2 cm) from the placental territory of each MC twin pregnancy. Regions with obvious calcification or infarction were avoided. The placental specimens were briefly rinsed with normal saline to remove the blood. The placenta tissue fragments were then divided into two horizontal segments: the basal plate (maternal side) and the chorionic surface (fetal side). Both the decidual layers along the basal plate as well as the chorionic surface and membranes were removed by sharp dissection and placental fragments were obtained at the middle of the initial placental depth. The middle placental tissues were kept in RNALater reagent (Thermo Fisher Scientific, Waltham, MA, USA) at 4 °C for 24–48 h. Subsequently, the tissues were transferred to new tubes and kept in a freezer at − 70 °C for long-term storage [15]. GLUT1, GLUT3 and HIF-1α gene expression were measured in each twin’s placental territory.

### Placental GLUT1, GLUT3 and HIF-1α gene expression analyses

#### RNA extraction and real-time qPCR

Total RNAs from placenta tissue were isolated with the TRIzol reagent (Invitrogen). First-strand cDNA for RT-qPCR was synthesized with cDNA reverse transcription kit (Applied Biosystems, Life Technologies, Carlsbad, CA, USA). The expression levels of GlUT1 were detected with forward primer, 5′- tatctgagcatcgtggccat-3′, and the reverse primer: 5′- aagacgtagggaccacacag-3′. The expression levels of GlUT3 were determined with the forward primer: 5′- tcctgggtcgcttggttatt-3′ and the following reverse primer: 5′- agggctgcactttgtaggat-3′.. The expression levels of HIF-1α were measured following reactions using the forward primer: 5′-TGCTC ATCAG TTGCC ACTTC-3’and reverse primer: 5′-TCCTC ACACG CAAAT AGCTG-3′. GAPDH mRNAs were analyzed with forward primer: 5′- ggtatcgtggaaggactcatgac-3′ and reverse primer: 5′-atgccagtgagcttcccgt-3′. QPCR conditions were as follows: initial denaturation step at 95 °C for 10 min, followed by 40 cycles of 95 °C for 15 s and 60 °C for 60 s, plus a final melt curve step using power SYBR® Green PCR master mix (Applied Biosystems, Life Technologies, Carlsbad, CA,USA). The relative expression level of each sample was normalized to the corresponding GAPDH. Data was calculated with a comparative Ct method by using 2-ΔΔCt formula. The placental GLUT1, GLUT3 and HIF-1α gene expression ratios for the two placental territories in a twin pair is presented as the fold changes between the two fetuses in MC twins: (relative GLUTs (HIF-1α) expression levels of the selective FGR twin)/ (relative GLUTs (HIF-1α) expression levels of the AGA twin).

### Data analysis and statistics

Data were analyzed using SPSS 11.0 statistical software (SPSS Inc., Chicago, IL, USA). Data are expressed as mean ± standard deviation, median [inter-quartile range] and frequency (%) as when appropriate. Qualitative data were compared by means of X2 test or Fisher exact test (when cells have expected count less than 5). Continuous variables were tested for normality. Two-sample Student t test or Mann-Whitney U test was used to compare between groups for the continuous variable. One sample t test with test value as 1.0 was applied to test the placenta GLUT1, GLUT3 and HIF-1α gene expression ratios between selective FGR and AGA fetuses. Two-tailed *p* values of < 0.05 were considered statistically significant.

## Results

15 MC twin pregnancies with selective FGR that met the inclusion criteria and that had placentas collected were selected for this study. The clinical characteristics of the 15 MC twin pregnancies with selective FGR according to the pattern of selective FGR twin’s UA Doppler were listed in Table 1. There were nine twin gestations with selective FGR twin displaying normal UA Doppler (group 1) and six twin pregnancies with selective FGR showing abnormal UA Doppler (group 2). The maternal age and gestational age at delivery, birth weight discordance (%) and birth weight of AGA and selective FGR twins were not significantly different between group 1 and 2 MC twin pregnancies.

**Table 1:** Characteristics of monochrionic twin with selective fetal growth restriction (FGR) with normal (group 1) and abnormal (group 2) selective FGR twin umbilical artery Doppler

	Group 1 (*n* = 9)	Group 2 (*n* = 6)	*P* value
Maternal age at delivery (year-old)	32.2 ± 3.6	33.2 ± 3.7	0.60
Gestational age at delivery (weeks)	33.0 ± 3.1	31.6 ± 4.5	0.52
Birth weight discordance (%)	37.4 ± 8.1	42.0 ± 11.2	0.34
Birth weight of AGA twin (g)	1876 ± 575	1564 ± 813	0.36
Birth weight of selective FGR twin (g)	1203 ± 474	970 ± 493	0.40

BW discordance = [(body weight of the AGA twin − body weight of the selective FGR twin)/ (body weight of the AGA twin)] × 100%

*GA* gestational age, *BW* birth weight, *FGR* fetal growth restriction, *AGA* appropriate for gestational age

The gene expression ratios of GLUT1, GLUT3 and HIF-1α in the 15 MC twin pregnancies with selective FGR are listed in Table 2. The gene expression of GLUT1, GLUT3 and HIF-1α between AGA and selective FGR placenta territories do not show significantly difference (one sample t test, test value set as1.0).

**Table 2:** The placenta GLUT1, GLUT3 and HIF-1α gene expressions between appropriate for gestational age (AGA) and fetal growth restriction (FGR) twin in the 15 monochorionic twins with selective FGR evaluated by gene expression ratio

	gene expression ratio	*P* value
GLUT1	0.97 ± 0.52	0.81
GLUT3	1.42 ± 0.90	0.09
HIF-1α	1.20 ± 0.56	0.18

*P* values were calculated by one sample t test and the test value was set as 1.0

The placental GLUT1, GLUT3 and HIF-1α gene expression ratios for the two placental territories in a twin pair is presented as the fold changes between the two fetuses in monochorionic twins: (relative GLUTs (HIF-1α) expression levels of the selective FGR twin)/ (relative GLUTs (HIF-1α) expression levels of the AGA twin)

*FGR* fetal intrauterine growth restriction, *AGA* appropriate for gestational age

The MC twin pairs expression of GLUT 1, GLUT3 and HIF-1α were separately analyzed in Group 1 (normal UA Doppler) and Group 2 (UA Doppler with AREDF). Comparison of normally grown and selective FGR twin placenta territories in Group 1 showed no difference in expression of GLUT1, GLUT3 and HIF-1α. Comparison in Group 2 reveals that GLUT 3 and HIF-1α gene expression are significantly up-regulated in selective FGR twins’ placental territory. (Table 3).

**Table 3:** GLUT1, GLUT3 and HIF-1α gene expression ratios between monochorionic twin pregnancies with selective FGR with (group2) and without (group1) selective FGR twin abnormal umbilical artery Doppler

	Group 1 twin pregnancies (*N* = 9)	*P* value of group 1 twin pregnancies	Group 2 twin pregnancies (*N* = 6)	*P* value of group 2 twin pregnancies
GLUT1 gene expression ratio	0.86	0.48	1.13	0.43
GLUT3 gene expression ratio	0.88	0.36	2.23	0.015
HIF-1α gene expression ratio	0.91	0.37	1.65	0.045

Data are expressed as median

An abnormal UA Doppler was defined as persistently absent or reverse end-diastolic flow

The placental GLUT1, GLUT3 and HIF-1α gene expression ratios for the two placental territories in a twin pregnancy pair is presented as the fold changes between the two fetuses in monochorionic twins: ((relative GLUTs (HIF-1α) expression levels of the selective FGR twin)/ (relative GLUTs (HIF-1α) expression levels of the appropriate for gestational age (AGA) twin))

*P* values were calculated by one sample t test and the test value was set as 1.0

*FGR* fetal growth restriction

## Discussion

This study evaluates the gene expression of placenta GLUT1, GLUT3 and HIF-1α in human MC twin pregnancies with selective FGR. When the selective FGR twin had normal antenatal Doppler studies, placental GLUT1, GLUT3 and HIF-1α gene expression are not significantly difference with the AGA twin (group1 twin pregnancies). But, when the selective FGR twin had abnormal Doppler studies; the GLUT3 and HIF-1α gene expressions were up-regulated in the selective FGR fetus’ placenta territory (group 2 twin pregnancies). Since GLUT3 and HIF-1α are both up-regulated in selective FGR fetus’ placental territory, HIF-1α may be responsible for the up-regulation of placental GLUT3 gene transcription as demonstrated in the BeWo choriocarcinoma model [13].

Janzen et al. reported that in late growth restriction, the placental GLUT3 protein had been reported as demonstrating increasing expression, but GLUT 1 and GLUT 4 protein expressions were reported as not being significantly altered [10]. We also found the GLUT1 gene expression were not different between AGA and selective FGR fetuses in both group 1 and 2 twin pregnancies in this study. GLUT3 expression in the placenta varies with gestational age [18]. But, since this was a MC twin study, both are delivered at the same gestational age and have similar genetics.

The placental perfusions of the two fetuses in MC twins with selective FGR are unequal, especially when abnormal UA Doppler are identified [19]. The cord blood glucose level was not significantly different between AGA and selective FGR fetuses in MC twin pregnancies with selective FGR as was noted in our previous findings [20]. Owing to a combination of these two findings, authors suspected that placenta GLUT3 gene up-regulation in selective FGR fetus in group 2 twin pregnancy was not stimulated by different glucose concentrations but rather due to differential perfusion. The impact of increased GLUT3 expression in FGR placenta on the maternal to fetal circulation is unclear. It had been explained as that increased GLUT3 expression may represent a mechanism by which the placenta attempts to increase glucose transport to the fetal circulation as an adaptive response in a hypoxic environment [10].

The weakness of this study is the presence of inter-twin anastomoses in MC twin pregnancy; the anastomoses may play a role in affecting the individual perfusion to the two fetuses though we had excluded TTTS and TAPS cases (those with significant inter-twin vascular imbalance) into this study. Monozygotic dichorionic twin with selective FGR could make a better model for this study since there was no inter-twin anastomosis identified, but it is hard to include enough such cases. Dizygotic twins with selective FGR are also without inter-twin anastomosis, but some genetic differences would still exist.

## Conclusion

The GLUT3 and HIF-1α gene expression were up-regulated in the placental territory of the selective FGR fetus compared to the territory of the AGA fetus in MC twin pregnancies with abnormal UA Doppler of FGR fetus, this gene expression difference was not found when the Doppler were normal. The authors suspect that the selective FGR fetus with abnormal UA Doppler experiences significant hypo-perfusion, than the fetus with normal UA Doppler. The hypo-perfusion leads to increased expression of GLUT3 which may be mediated by up-regulation of HIF-1α.

## Data Availability

The datasets obtained and/or analyzed during the current study are available from the corresponding author on reasonable request.

## Abbreviations

GLUTs: Glucose transporters
HIF-1α: hypoxia-inducible factor-1α
MC: Monochorionic
FGR: Fetal growth restriction
AGA: Appropriate for gestational age
UA-AREDF: Umbilical artery-absent or reverse end-diastolic flow
TAPS: Twin anemia–polycythemia sequence
TTTS: Twin–twin transfusion syndrome
